# Energy gap and aromatic molecular rings

**DOI:** 10.1098/rsos.231533

**Published:** 2024-04-03

**Authors:** Ali K. Ismael, Alaa Al-Jobory

**Affiliations:** ^1^ Department of Physics, Lancaster University, Lancaster LA1 4YB, UK; ^2^ Department of Physics, College of Education for Pure Science, Tikrit University, Tikrit, Salah Al Deen 34001, Iraq; ^3^ Department of Physics, College of Science, University of Anbar, Al Rumadi, Al Anbar 31001, Iraq

**Keywords:** energy gap, density functional theory, gap, aromatic, molecular, rings

## Abstract

The manuscript combines rational density functional theory simulations and experimental data to investigate the electrical properties of eight polycyclic aromatic hydrocarbons (PAHs). The optimized geometries reveal a preference for one-row, two-row and three-row ring distributions. Band structure plots demonstrate an inverse correlation between the number of aromatic rings and band gap size, with a specific order observed across the PAHs. Gas phase simulations support these findings, though differences in values are noted compared to the literature. Introducing a two-row ring distribution concept resolves discrepancies, particularly in azulene. The B3LYP function successfully bridges theoretical and experimental gaps, particularly in large PAHs. The manuscript highlights the potential for designing electronic devices based on different-sized PAHs, emphasizing a multi-ring distribution approach and opening new avenues for practical applications.

## 1. Introduction

Aromaticity is an essential concept in organic chemistry. This property is commonly used to discuss the unique stability of cyclic π-conjugated molecules [1]. Huckel’s rule [2] states that planar π-conjugated cyclic molecules with (4n+2)π electrons are stabilized (i.e. aromatic), whereas those with 4nπ electrons are not stabilized (i.e. anti-aromatic). Many theoretical approaches including valence bond analysis [3,4], graph theory [5,6] and molecular orbital theory [4,7] support Huckel’s rule [8–10]. Experimental studies involving nuclear magnetic resonance peak shifts owing to the presence of ring currents and carbon–carbon bond distance analysis also support this rule. To stabilize aromatic or anti-aromatic molecules, there are several methods, one of which is π-stacking. One theoretical study suggested that the stability orientation of cyclobutadiene bilayers that are stacked in a face-to-face form is at a π-stacking distance of 2.45 Å [11–13]. Further, the aromatic intermediate regions were demonstrated to exist in the reaction pathway from the cyclobutadiene dimer to cubane [14]. The stacked aromaticity, which is defined as the stacking of anti-aromatic molecules, was explored via a thorough theoretical analysis of graph and ring current theories [15,16]. On the other hand, stacked aromaticity experimental investigations were limited to a few studies, including [17], where it was demonstrated that meso-isopropyl norcorrole (NC) does not exhibit π-stacking even in a concentrated solution or crystalline phase, even though the micellar capsules display high stability towards heating and concentration change. While Kawashima *et al*. [18] proved the NC in solution, it forms a twist stacking arrangement with effective interplanar orbital overlap and exists in equilibrium between stacked and unstacked structures. The same study [18–20] also determined the highest occupied molecular orbital and lowest unoccupied molecular orbital energy levels of the slipped and twisted stacks of the NC monomer and estimated the energy gaps for three different NC cyclophanes using the B3LYP/def2-TZVP function.

**Figure 1.**
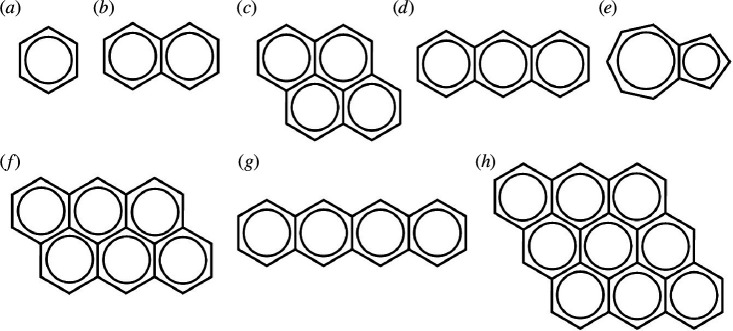
Illustration of an oligoacene series including benzene (1R), naphthalene (2R), pyrene (4R), anthracene (3R), azulene (2R) (*a*–*e*), and PAHs, formation as clusters of anthracene molecules anthanthrene (6R), tetracene (4R) and dibenzocoronene (9R) (*f–h*). R refers to the NAR in each PAH.

In the current study, we investigate the electronic properties of an oligoacene series including benzene, naphthalene, azulene, anthracene, pyrene, tetracene and polycyclic aromatic hydrocarbons (PAHs), which form clusters of anthracene molecules as shown in figure 1. The major investigation here is dedicated to the electronic structure properties including optimization, energy levels, degeneracy states, band structures, energy gap analyses and conducting differences. These parameters have a significant effect on the electric and thermoelectric transport of PAHs. By exploring the parameters mentioned above, it might be possible to answer whether aromatization influences the electronic properties of PAHs.

## 2. Computational methods

All the theoretical simulations were carried out by using the density functional theory (DFT) code SIESTA [21]. It is well established that the DFT method is very successful in studying different materials [22–28]. The optimum geometries of isolated PAH components were obtained by relaxing the PAHs until all forces on the atoms were less than 0.01 eV Å^–1^ (for more details, see optimized DFT isolated structures and electronic supplementary material, figure S1). We used a double-zeta plus polarization orbital basis set, norm-conserving pseudopotentials, the local density approximation (LDA) exchange correlation functional, and to define the real space grid, an energy cut-off of 250 Rydbergs. We also computed results using generalized gradient approximation (GGA) and found that the resulting functions were comparable [29–31] with those obtained using LDA. However, the (GGA-Perdew-Burke-Ernzerhof (PBE)) of the exchange and correlation functionals was used in this study. To provide more reliable validation for the presence of multi-aromatic rings, it is essential to evaluate our simulations using multiple methods. Therefore, calculations were carried out here also using the B3LYP functional [32] and the 6-311+G** basis set [33], implemented in the Gaussian9 program package [34].

## 3. Results and discussion

To understand how the use of a different size of PAHs affects the energy gap $E_{g}$ (i.e. conductivity), the electronic properties of single and multi-aromatic rings were modelled using a combination of DFT and quantum transport theory.

**Figure 2.**
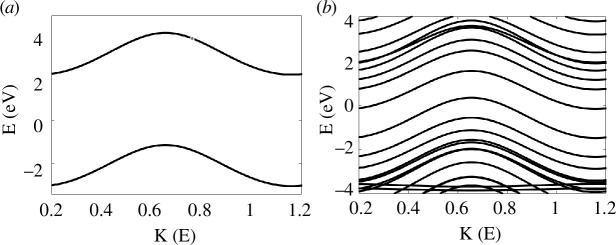
DFT band structures of single- and multi-ring molecules. Benzene 1R (*a*) and dibenzocoronene 9R (*b*). Other band structures are shown in the electronic supplementary material, figure S2.

As a first step towards understanding the electronic properties of PAH, we calculated the band structures for the studied molecules (figure 1). Figure 2*a* illustrates the band gap of the smallest aromatic molecule (benzene), which is one ring (1R). The figure estimates the gap to be 3.25 eV, and this value decreases to 1.51, 0.78, 0.42, 0.39 and 0.18 eV for naphthalene, pyrene, anthracene, azulene and anthanthrene, respectively, and the band gap disappears for tetracene and dibenzocoronene (for more details, see the electronic supplementary material, figures S2 and S3, and S2).

**Figure 3.**
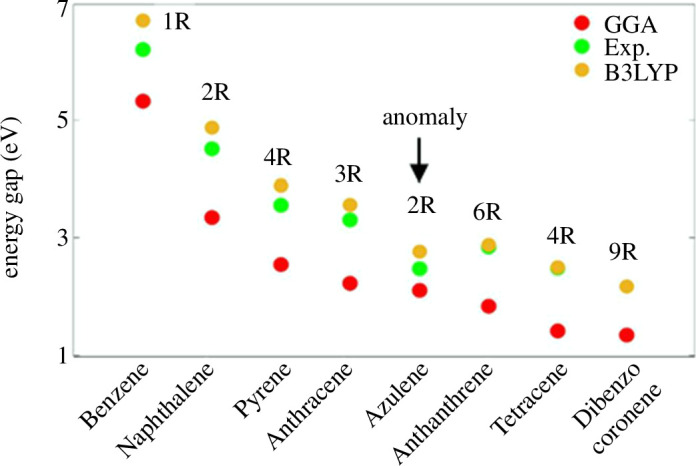
Energy gap ( $E_{g}$ ), as a function of different sizes of PAHs, molecules including benzene (1R), naphthalene (2R), pyrene (4R), anthracene (3R), azulene (2R), anthanthrene (6R), tetracene (4R) and dibenzocoronene (9R). GGA calculated the energy gap (red circles), experimentally measured the energy gap (green circles), and B3LYP calculated the energy gap (orange circles). R refers to NAR in each PAH.

Figure 2 and the electronic supplementary material, figures S2 and S3, evaluate the band gaps in periodic systems. These figures elucidate an inverse correlation between the number of aromatic rings (NAR) and band gap size. We now repeat the same calculations, however, in the gas phase (i.e. isolated system), for the eight PAHs. Simulations begin with the GGA-PBE. Figure 3 presents the energy gaps in eV units for the studied molecules. In general, the red circles demonstrate an inverse relationship between the NAR and energy gap ( $E_{g}$ ), which supports the band structure calculations. Although the smallest molecule (benzene) possesses the largest gap and the biggest molecule (dibenzocoronene) has the smallest gap, the value of $E_{g}$ depends on the molecule details. For example, the energy gap of tetracene is expected to be larger than anthanthrene as the former consists of four aromatic rings (4R), while the latter is 6R. Similarly for anthracene (3R) and pyrene (4R).

To examine the validity of our DFT simulations on the electronic properties of aromatic molecular rings, we shall check them against the literature [35–39]. The green circles show the experimental $E_{g}$ for seven moieties (note: no measurement for dibenzocoronene, see the electronic supplementary material, table S1). Experimental measurements confirm that GGA function successfully predicts the energy gaps for the studied PAHs. Notwithstanding that simulations and measurements agree, some of the aromatic molecules show unexpected behaviour such as $E_{g}\left(6R\right)$ > $E_{g}$ (4R) and $E_{g}\left(4R\right)$ > $E_{g}$ (3R) (i.e. anthanthrene > tetracene and pyrene > anthracene). To gain a deeper insight into the electronic properties of these aromatic moieties, we shall consider the distribution of the rings within the moiety in addition to the number of rings.

To accommodate this point, we compare pyrene, which has four rings distributed in two rows, against anthracene, which has three rings distributed in one row. Experiment and theory gaps (green and red circles) suggest that the two-row ring distribution yields a larger energy gap than the one-row distribution despite the fact that the former distribution involves more rings than the latter (i.e. 4 rings versus 3). Similarly, for anthanthrene versus tetracene (i.e. $E_{g}\left(6R\right)$ > $E_{g}$ (4R)). Notably, the two-row distribution approach applies successfully even on the same size moieties (same number of rings), as illustrated by the energy gaps of pyrene and tetracene (3.5 and 2.5 eV). Furthermore, the experimental energy gap of the azulene molecule appears to be significantly small for such a two-ring molecule. It is unexpected for the energy gap of azulene to be lower than that of anthracene and pyrene (i.e. $E_{g}\left(2R\right)$ < $E_{g}$ (3R) and $E_{g}\left(2R\right)$ < $E_{g}$ (4R)).

Surprisingly, the gap of azulene is even lower than that of naphthalene, which possesses the same number of rings (2R). We attribute this to the fact that the azulene molecule differs from the rest by the size of its rings. Strictly speaking, all PAH moieties comprise six-membered rings, while azulene encompasses five- and seven-membered rings (figure 1*e*). The effect of unequal rings (five- and seven-membered rings) is evidently reflected in the measured energy gap of azulene, as it is lower than any measured molecule in this study even though it has only 2Rs (i.e. anomaly, $\ll \left(E_{g}\right)Exp.$ ), as shown in figure 3.

Despite the fact that the GGA function predicts the correct order of the energy gaps for seven PAHs, it fails in the case of azulene and the difference between the experiment and the GGA gaps is remarkably high (red against green circles) compared to other DFT functions.

To perform a sufficiently good comparison, we shall repeat the energy gap calculations using the B3LYP functional. The energy gap determined by the B3LYP function manifests the accuracy of this function against the experiment (orange versus green circles). Thus, the theory−experiment difference shrinks pronouncedly and completely vanishes in large PAHs such as tetracene (4R) and anthanthrene (6R).

## 4. Conclusions

In summary, through rational DFT simulations examined by experimental measurements, we have demonstrated that the electrical properties of eight PAHs can be modulated by varying NAR and their locations/orientations within the aromatic moieties. Fully optimized geometries suggest that aromatic molecules energetically prefer to construct one-row, two-row and three-row ring distributions.

Band structure plots in periodic systems suggest an inverse correlation between the NAR and the band gap size ( $E_{g}$ ). These simulations suggest that the band gaps follow the order $E_{g}\left(benzene\right)$ **>** $E_{g}\left(naphthalene\right)$ **>** $E_{g}\left(pyrene\right)$ **>** $E_{g}\left(anthracene\right)$ **>** $E_{g}\left(azulene\right)$ **>** $E_{g}\left(anthanthrene\right)$ **>** $E_{g}\left(tetracene\right)$ **>** $E_{g}\left(dibenzocoronene\right)$ . The same calculations were repeated, however, in the gas phase for the eight PAHs, and the same trend was obtained. To validate the GGA-PBE energy gap simulations, we examined them against the literature, and in general, they agree in the order (except for azulene), but with a large difference in the values.

Two-row ring distribution concept was introduced to solve the discrepancy of the inverse relationship between the NAR and energy gap. For instance, $E_{g}\left(6R\right)$ > $E_{g}$ (4R) and $E_{g}\left(4R\right)$ > $E_{g}$ (3R) (i.e. gap of anthanthrene > tetracene and pyrene > anthracene). This approach successfully cleared up the disparity and the azulene energy gap anomaly (i.e. $\ll \left(E_{g}\right)Exp.$ ), which was satisfied by the unequal ring size effect (five- and seven-membered rings). B3LYP function bridged the gap difference between theory and experiment until zero in large PAHs such as tetracene (4R) and anthanthrene (6R). Additionally, this study opens new ideas for designing electronic devices based on using different size of PAHs, focusing on a multi-ring distribution approach, with potential practical applications.

## Data Availability

Data is available in the electronic supplementary material [40].
